# Scaphoid Stress Fracture in High-Level Gymnast: A Case Report

**DOI:** 10.1155/2011/492407

**Published:** 2011-09-26

**Authors:** J. C. Nakamoto, M. Saito, G. Medina, B. Schor

**Affiliations:** Instituto Vita, R. Mato Grosso, 306 No. 1 Andar, 01239-040 São Paulo, SP, Brazil

## Abstract

We present the case of an 18-year-old high-level gymnast who sustained a stress fracture of the scaphoid associated with a distal radial epiphysiolysis. Clinical evaluation demonstrated decreased range of motion of the affected wrist and insidious pain on the snuffbox and tenderness on the distal radial physis. He was submitted to surgical treatment with scaphoid percutaneous fixation and radial styloid process in situ fixation. Clinical features improved, and he got back to competition 6 months after surgery without symptoms and with complete range of motion.

## 1. Introduction

Lower extremity stress fractures are common and are usually related to overtraining. However, this kind of injury is rare in the upper limbs and there are few reports in the literature regarding this trauma [1].

Scaphoid fractures usually occur in young adults after a fall on the outstretched arm resulting in acute dorsiflexion of the wrist. The patient presents with pain at the radial side of the wrist, tenderness on the snuffbox, pain on palpation of the scaphoid tubercle, and difficulties with range of motion. Radiographs are necessary to confirm the diagnosis, and sometimes a magnetic resonance imaging must be performed [2, 3].

Patients who perform activities with repetitive stress on the wrist and present similar complaints, although without a history of acute trauma, should be thoroughly investigated and one should suspect of a scaphoid stress fracture [4, 5].

Chronic repetitive dorsiflexion with axial compression of the wrist are frequently associated with injuries of the distal radial growth plate. This injury is typically seen on radiographs as a widened and irregular distal radius physis [6, 7].

Although wrist pain in gymnasts has been considered a “normal and direct consequence of the sport,” [2] the complaint of pain in these younger athletes must be carefully evaluated. 

We present the case of a gymnast who sustained a stress fracture of the scaphoid associated with a distal radial epiphysiolysis.

## 2. Case Report

An 18-year-old gymnast complained of insidious-onset and increasing right-wrist pain. He had been practicing gymnastics six hours a day, three days a week, for the past seven years.

Approximately three months before his visit, he began to experience severe pain which worsened with wrist extension, especially when tumbling on the pommel horse or on the parallel bars.

Initially, he thought he had a muscle strain and treated it conservatively with ice packing and decreasing his activity level, but there was little improvement. There was no previous history of pain or trauma on the affected wrist.

On physical examination the patient had soft callus over the distal wrist and the right scaphoid. He reported pain in the anatomic snuffbox and in distal radial epiphysis. Wrist extension was 38° on the right and 60° on the left. Patient's range of motion was limited to 38° of extension and 12° of radial deviation on the right wrist but presented no flexion or ulnar deviation limitations. Grip strength was 46.5 Kgf on the right hand and 81.5 Kgf on the left hand. Pronation, supination, and digital motion were within normal limits. 

**Figure 1:**
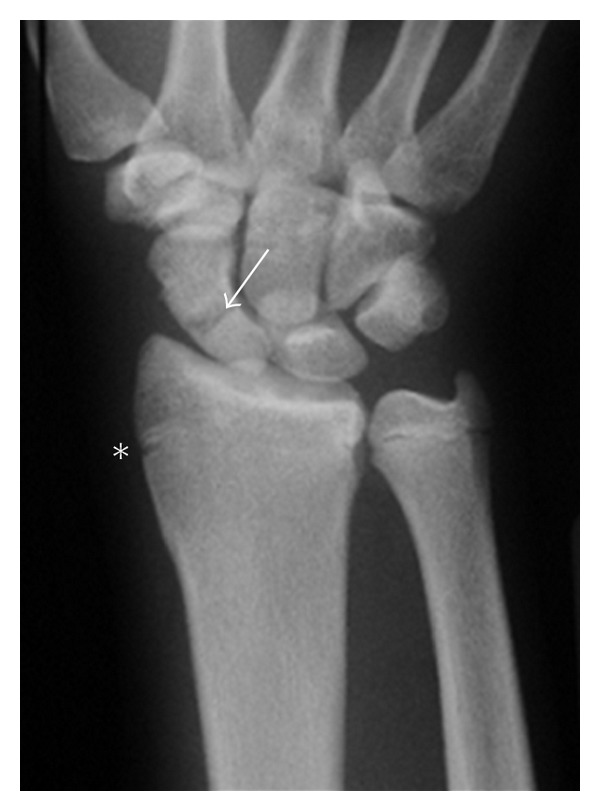
Plain coronal radiograph suggesting fracture at the waist of the scaphoid (arrow) and widening of the radial aspect of the distal radial epiphysis (∗).

**Figure 2:**
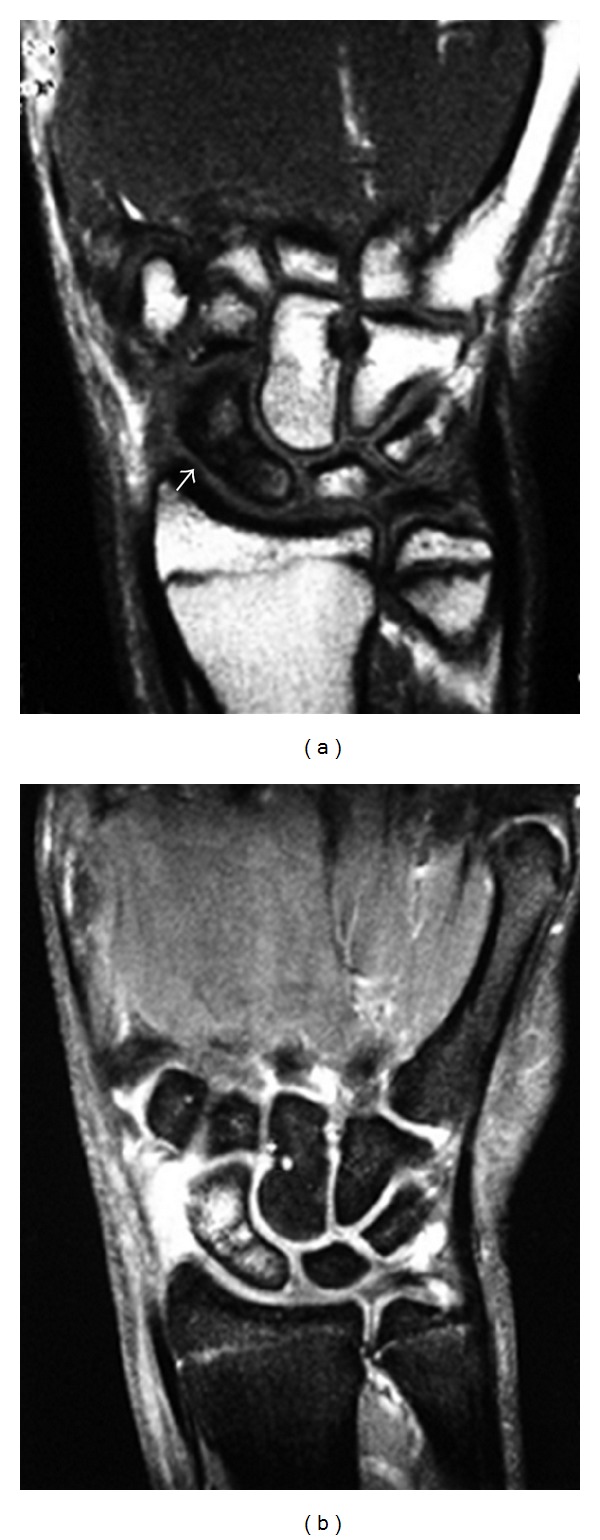
(a) T1-weighted coronal magnetic resonance image showing incomplete fracture at the waist of the scaphoid (white arrow); (b) T2-weighted coronal magnetic resonance image showing bone edema of the proximal and distal poles.

A posteroanterior (PA) plain radiograph of the wrist showed a fracture at the waist of the scaphoid and widening of the radial aspect of the distal radial epiphysis (Figure 1). A magnetic resonance imaging (MRI) examination of the wrist was performed and revealed an incomplete fracture at the waist of the scaphoid and a bone marrow edema in the proximal and distal poles (Figure 2). 

**Figure 3:**
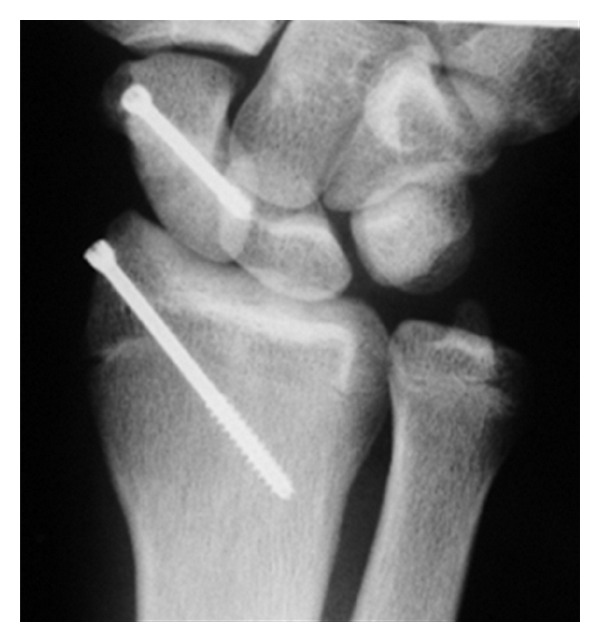
Plain PA radiograph shows the percutaneous fixation and radial styloid process in situ fixation 8 weeks after operation.

We decided on operative treatment with scaphoid percutaneous fixation and radial styloid process in situ fixation (Figure 3).

In the first week postoperatively the patient used a short orthosis that immobilized the metacarpophalangeal joint of the thumb and rehabilitation began aiming to control the edema and increase range of motion. After 8 weeks, radiographs showed signs of consolidation, and at physical examination the wrist presented 52° extension, 62° flexion, 12° radial deviation, and 20° ulnar deviation. Grip strength was 66 Kgf in the right wrist (Table 1).

In the twelfth week the patient was allowed to progressively return to physical activities and, within 16 weeks, he returned to activities on apparels. 

At the final evaluation, six months after operation, the athlete was competing without complaints.

## 3. Discussion

Wrist pain is a very common symptom in gymnasts. Dobyns and Gabel reported that 88% of gymnasts have experienced wrist pain during activity and 80 to 90% of the injuries are secondary to constant mechanical overload [8].

The scaphoid is the most fractured carpal bone, but its reduced size and irregular shape makes diagnosis very difficult. Due to the vascularization of the scaphoid, fractures may jeopardize bone nutrition and lead to pseudoarthrosis or avascular necrosis. Proximal pole vascularization is dependent on the vessels that cross the waist. 

Scaphoid stress fracture is relatively rare, with few reports in the literature [1–5]. Fractures combined to traumatic epiphysiolysis are even rarer. 

Before symptoms onset, our athlete performed repetitive activities which reproduced the acute fracture mechanism. These activities were performed in high intensity due to the competition calendar. Nevertheless, there are no records of major trauma that could have triggered the symptoms. The insidious onset of pain eliminates the possibility of a misdiagnosed acute fracture, and the presence of bone marrow edema throughout the bone, as seen in our patient, is also indicative of a stress fracture.

The concomitant physeal injury seen in radiographs and MRI presented as a complicating factor of the scaphoid fracture, requiring greater attention to the wrist.

Weiker proposed that wrist injuries in gymnasts occur from wrist and fingers muscles weakness [5]. This weakness prevents an adequate impact force damping during wrist dorsiflexion movements, hereby, causing impact on distal radius and carpal bones. The volar ligaments of the carpus protect the proximal pole of the scaphoid while its proximal portion remains exposed to the impact. The weaker portion of this bone is its waist, located immediately distal to the volar ligaments and, therefore, the most common site of injury [5] and may be prone to stress fractures. 

Gymnast's chronic wrist pain, even without acute trauma history, should be suspect of a possible scaphoid stress fracture. Radiograph is the initial trial exam, although, sometimes, it may not be able to show the injury. When suspected, the MRI is the gold standard. Even the scintigraphy may not be conclusive, as reported by Hanks et al. [2]. 

The reasons for treating the scaphoid and distal radius operatively were (a) the possibility of an absolute stabilization of the fracture that could prevent further risk of deviation, and, consequently, prevent from a more complex operative procedure and (b) earlier rehabilitation with increased range of motion and intrinsic and extrinsic muscle strengthening. 

Rehabilitation was performed by a specialized hand therapist who aimed to optimize it and allow an earlier return to sport activities. 

## Figures and Tables

**Table 1:** Range of motion and grip strength on the right wrist and hand before and after treatment.

Parameter	Before surgery	After surgery
Extension	38°	52°
Flexion	62°	62°
Radial deviation	12°	12°
Ulnar deviation	20°	20°
Grip strength	46.5 Kgf	66 Kgf
